# Evidence of altered cardiac autonomic regulation in myalgic encephalomyelitis/chronic fatigue syndrome: A systematic review and meta-analysis

**DOI:** 10.1097/MD.0000000000017600

**Published:** 2019-10-25

**Authors:** Maximillian J. Nelson, Jasvir S. Bahl, Jonathan D. Buckley, Rebecca L. Thomson, Kade Davison

**Affiliations:** a Alliance for Research in Exercise, Nutrition and Activity (ARENA), University of South Australia; b Adelaide Medical School and Robinson Research Institute, University of Adelaide, Adelaide, Australia.

**Keywords:** autonomic dysfunction, chronic fatigue syndrome, fatigue, heart rate, myalgic encephalomyelitis

## Abstract

**Background:**

Myalgic Encephalomyelitis/Chronic Fatigue Syndrome (ME/CFS) is a complex condition with no reliable diagnostic biomarkers. Studies have shown evidence of autonomic dysfunction in patients with ME/CFS, but results have been equivocal. Heart rate (HR) parameters can reflect changes in autonomic function in healthy individuals; however, this has not been thoroughly evaluated in ME/CFS.

**Methods:**

A systematic database search for case-control literature was performed. Meta-analysis was performed to determine differences in HR parameters between ME/CFS patients and controls.

**Results:**

Sixty-four articles were included in the systematic review. HR parameters assessed in ME/CFS patients and controls were grouped into ten categories: resting HR (RHR), maximal HR (HR_max_), HR during submaximal exercise, HR response to head-up tilt testing (HR_tilt_), resting HR variability (HRV_rest_), HR variability during head-up tilt testing (HRV_tilt_), orthostatic HR response (HR_OR_), HR during mental task(s) (HR_mentaltask_), daily average HR (HR_dailyaverage_), and HR recovery (HRR) Meta-analysis revealed RHR (MD ± 95% CI = 4.14 ± 1.38, *P* < .001), HR_tilt_ (SMD ± 95% CI = 0.92 ± 0.24, *P* < .001), HR_OR_ (0.50 ± 0.27, *P* < .001), and the ratio of low frequency power to high frequency power of HRV_rest_ (0.39 ± 0.22, *P* < .001) were higher in ME/CFS patients compared to controls, while HR_max_ (MD ± 95% CI = –13.81 ± 4.15, *P* < .001), HR at anaerobic threshold (SMD ± 95% CI = –0.44 ± 0.30, *P* = 0.005) and the high frequency portion of HRV_rest_ (–0.34 ± 0.22, *P* = .002) were lower in ME/CFS patients.

**Conclusions:**

The differences in HR parameters identified by the meta-analysis indicate that ME/CFS patients have altered autonomic cardiac regulation when compared to healthy controls. These alterations in HR parameters may be symptomatic of the condition.

## 1 Introduction

Myalgic Encephalomyelitis/Chronic Fatigue Syndrome (ME/CFS) is a condition with complex aetiology and is commonly characterised by debilitating fatigue that is not resolved with rest, with additional symptoms including muscle and joint pain, tender lymph nodes, sore throat, and cognitive difficulties.^[2]^ Despite numerous hypotheses being proposed to explain the pathology of ME/CFS, there is a lack of conclusive evidence regarding the pathophysiology underlying this condition and no reliable clinical marker of ME/CFS exists.^[3]^ As a result, diagnosis of ME/CFS has relied on patients meeting one of a range of consensus based diagnostic criteria, the most widely used being the Centers for Disease Control and Prevention (CDC) 1994 criteria.^[1]^ The CDC 1994 criteria requires the presence of unexplained persistent or relapsing chronic fatigue which is of new or definite onset, and results in a substantial reduction in prior levels of occupational, educational, social or personal activities. In addition, to meet the diagnosis the person must also display at least 4 of the following secondary symptoms which must not have predated the fatigue:

1. impairment in short-term memory or concentration;
2. sore throat;
3. tender cervical or axillary lymph nodes;
4. muscle or multi-joint pain;
5. headache;
6. unrefreshing sleep;
7. post-exertional malaise that remains for a period of more than 24 hours.

More recently, other diagnostic criteria have emerged which have increased the emphasis on the importance of post-exertional malaise to help differentiate ME/CFS from other conditions with similar symptoms (e.g., Fibromyalgia).^[3,4]^ Estimates of the prevalence of ME/CFS range from 0.2%^[5]^ to 6.4%^[6]^ in the developed world, with the variation in estimates likely due to differences in the diagnostic criteria applied.^[7]^ Regardless of the prevalence, ME/CFS is a complex condition, for which diagnosis remains difficult. Accordingly, the identification of a reliable marker of ME/CFS remains highly desirable.

Evidence of altered autonomic nervous system (ANS) regulation of cardiovascular function has been observed in patients with ME/CFS.^[8,9]^ Patients commonly present with concurrent cardiovascular conditions such as postural orthostatic tachycardia syndrome (POTS),^[10]^ and disturbances in additional markers of ANS function including blood pressure variability, and altered responses to head-up tilt testing (HUTT).^[11–13]^ However, these studies have used inconsistent methodologies and provided variable results, making it difficult to conclusively establish the nature and extent of any derangement of cardiovascular autonomic regulation.

One approach to assessing cardiovascular autonomic regulation which has commonly been used in ME/CFS patients is the assessment of a range of heart rate (HR) parameters such as HR variability (HRV),^[14]^ HR recovery (HRR),^[15]^ and HR acceleration.^[16,17]^ A recent meta-analysis confirmed the ability of some HR markers of cardiac autonomic regulation to identify when athletes have become fatigued from too much exercise,^[18]^ but there has been no systematic evaluation of whether these parameters are altered following other types of fatigue, including in patients whose fatigue originates from ME/CFS. The purpose of the present study was to systematically review, and meta-analyse, the literature reporting markers of cardiac autonomic regulation in patients with ME/CFS to determine whether there were differences in HR parameters between patients and controls. This was done in an effort to determine whether any markers of cardiac autonomic dysfunction might be useful to aid in the diagnosis of ME/CFS.

## 2 Methods

This systematic review was performed in accordance with the Preferred Reporting Items for Systematic reviews and Meta-Analyses (PRISMA).^[19]^ The protocol for the review was registered with the International Prospective register for Systematic Reviews (PROSPERO; registration no. CRD 42016036731). As this study was a review of published literature, no ethics board approval was required.

### 2.1 Search strategy

The search strategy was formulated using the PICO (Population, Intervention, Comparator, Outcome) framework, and in consultation with an academic librarian.^[20]^ To be included in the present meta-analysis, articles had to report on studies which reported assessing heart rate parameters (O) in patients diagnosed with ME/CFS (P) compared to healthy controls (C), with intervention (I) not being relevant to the current review and meta-analysis. In addition, the study design was limited to clinical case-control studies (S).

### 2.2 Database searching

A literature search was performed using MedLine, Embase, SportDiscus, CINAHL, Scopus and AMED with no date restrictions. Literature searches were performed by 2 authors (MJN and JSB) on the same day in January 2017. A mixture of keywords and MeSH headings where appropriate, linked with the appropriate Boolean operators was used to identify relevant articles. The complete search strategy was (‘*Chronic Fatigue Syndrome*’ OR ‘*CFS*’ OR ‘*Myalgic Encephalomyelitis*’ OR ‘*ME*’) AND (‘*Heart rate*’ OR ‘*Heart rate variability*’ OR ‘*HRV*’ OR ‘*Heart rate recovery*’ OR ‘*HRR*’ OR ‘*Resting heart rate*’ OR ‘*RHR*’ OR ‘*Maximal heart rate*’ OR ‘*HRmax*’ OR ‘*Rate of heart rate increase*’ OR ‘*rHRI*’ OR ‘*Pulse rate*’). Reference lists of included articles were manually searched to identify additional articles. To ensure repeatability, agreement was required from the 2 relevant reviewers on the number of articles retrieved before proceeding.

### 2.3 Study selection

Articles were eligible for inclusion when the following criteria were fulfilled:

1. participants were adults (aged ≥ 18 years);
2. participants were diagnosed with ME/CFS based on recognised criteria: CDC 1988,^[2]^ CDC 1994,^[1]^ International Consensus Criteria (ICC),^[4]^ Canadian Consensus Criteria (CCC),^[3]^ or Oxford^[21]^);
3. studies included healthy control comparison group;
4. HR parameters were measured and reported, including, but not limited to: resting HR (RHR), maximal HR (HRmax), HRV, HRR and steady state exercise HR (HR_steadystate_);
5. data were adequately reported (mean and standard deviation (SD) or standard error (SE) or 95% confidence intervals (CI));
6. the article was either written in English or had a detailed English summary available;
7. ME/CFS patients were free of comorbid conditions (Fibromyalgia, Diabetes, etc); and
8. ME/CFS patients were not reported to be taking HR altering medication (β-blockers, etc). In the case of data being inadequately reported, the corresponding author was contacted with a request for additional data. Citations retrieved from the searches were uploaded on an online systematic review platform (Covidence systematic review software, Veritas Health Innovation Ltd., Melbourne, Australia).

Citations retrieved from the search were independently screened by 2 reviewers (MJN and JSB) and any conflicts were resolved by discussion, or referred to a third reviewer (KD) if consensus was not reached. Titles that met the eligibility criteria were then retrieved as full manuscripts and reviewed independently by 2 reviewers (MJN and JSB). Conflicts were resolved using the same process as from the initial screening stage.

### 2.4 Critical appraisal

Critical appraisal of included studies was performed to assess the validity of the included studies using the Joanna Briggs Institute ‘Checklist for Case Control Studies’.^[22]^ Assessment was independently performed by two separate authors (MJN and JSB) who were blinded to the others evaluations. Disagreement between reviewers was resolved through discussion and via consultation with a third author (KD) if required. Articles were scored on description of the groups, exclusion of selection bias, detection and statistical methods, equal exposure and accounting for confounding factors. Articles were scored as either ‘yes’, ‘no’, ‘unclear’ or ‘NA’ for all questions, with each article being assessed on ten questions. Inter-rater agreement for each item of the risk of bias tool was evaluated using the Kappa (κ) statistic.

### 2.5 Data extraction and analysis

Information from each study was collected on publication details, participant characteristics and numbers, diagnostic criteria used for ME/CFS patients, and results of HR parameter assessment. All HR variables were extracted and where a HR variable was reported by more than one study, data were pooled to compute the between group differences in patients and controls. Random-effects meta-analysis was performed using Review Manager (RevMan, version 5.3, Cochrane Collaboration, Oxford, UK), using the inverse variance method. Due to the consistency of methods used for measurement of HR_max_ and RHR, these parameters were expressed as the mean difference (MD) between groups ± 95% CI. All other data were expressed as the standardised mean difference (SMD) ± 95% CI, calculated by standardizing the mean difference between ME/CFS patients and controls by the pooled standard deviation from both patients and controls. Where data were reported separately based on gender or level of disease severity, mean and SD were pooled giving single values for ME/CFS patients and controls, rather than being reported separately. The presence of statistical heterogeneity was assessed through the *I*^2^ statistic. Statistical significance for all outcome measures was set to *P* < .05.

## 3 Results

**Figure 1:**
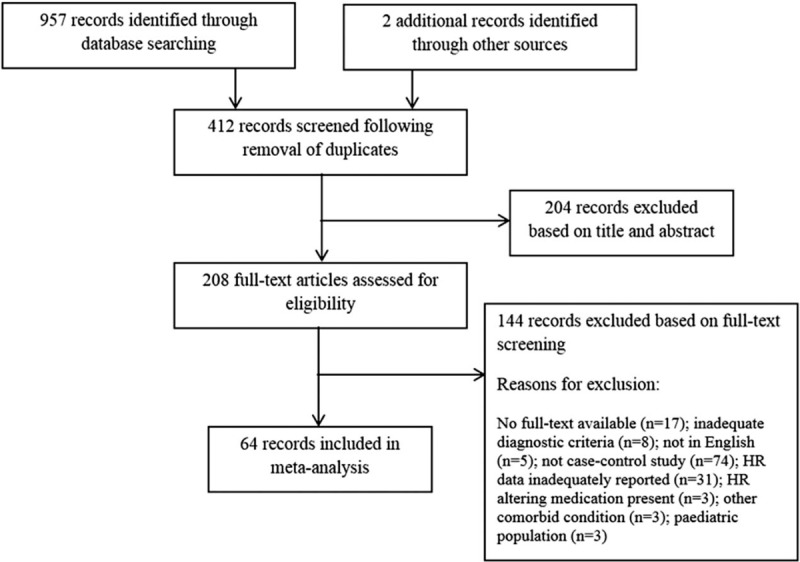
Literature search flow chart. *n* number of included studies.

The literature search initially identified 412 unique records. The 317 studies did not meet the inclusion criteria and were excluded. Of the remaining 95 studies, data were inadequately reported in 31. None of the authors provided missing data upon request, resulting in a total of 64 studies for inclusion. A summary of the search outcomes, including the number of studies excluded at various phases is shown in Figure 1.

### 3.1 Critical appraisal of included studies

Inter-rater agreement for critical appraisal was high (κ = 0.75). Critical appraisal revealed that the majority of included studies were of moderate or good quality. Seventeen articles were found to be of ‘moderate’ quality (score between 50% and 59%), while 30 studies were found to be of ‘good’ quality (score between 60% and 69%). Four articles were found to be of ‘low’ quality (score below 50%), with each article scoring 45%. Thirteen articles were found to be ‘high’ quality (score over 70%) The most common questions which resulted in a response of ‘No’ during critical appraisal were “Were confounding factors identified?” (performed in 20% of studies), and “Were strategies to deal with confounding factors stated?” (23%). Few studies (30%) reported on the specific length of exposure to the condition so it was difficult to assess the question “Was the exposure period of interest long enough to be meaningful?”. However, as this review only included studies which diagnosed ME/CFS through accepted criteria, and all of these criteria require the presence of the condition for at least 6 months, it is likely that all included studies reported on a meaningful exposure. That said, studies which did not explicitly report on the length of exposure to the condition were graded as “not available” for that question. Generally, patients and controls within the included studies were comparable, and both the exposure to the condition and all relevant HR parameters were assessed in a standard, valid and reliable manner. Appropriate statistical analysis were used in the vast majority of studies, however statistical analyses were unclear in 7 studies.^[23–29]^

### 3.2 Participants

In total, the 64 included studies reported on 2286 ME/CFS patients and 1758 healthy controls who had HR parameters assessed. Of these studies, 14 recruited exclusively female participants, and 50 studies recruited a mixed gender sample. Overall, 79% of ME/CFS patients were female (n = 1803), compared to 73% for controls (n = 1283), while 1 study^[23]^ did not specify the gender of participants.

### 3.3 Diagnostic criteria

The 1994 CDC criteria were the most commonly used diagnostic criteria^[1]^ (47 studies), followed by the 1988 CDC criteria^[2]^ (8 studies), the Oxford criteria^[21]^ (5 studies), and the ICC^[4]^ (5 studies). Four studies employed the CCC, in each case in addition to another form of criteria (CDC alone,^[30–32]^ CDC+ICC^[33]^).

### 3.4 Study outcomes

Overall, HR parameters extracted from the included studies were sorted into 10 categories, and included studies for each parameter are presented in Table 1    – resting HR (RHR), maximal HR (HR_max_) HR during submaximal exercise, heart rate during HUTT (HR_tilt_), Orthostatic HR response (HR_OR_), resting HRV (HRV_rest_), HRV during HUTT (HRV_tilt_), HR during mental task (HR_mentaltask_), daily average HR (HR_dailyaverage_), and HRR

**Table 1:**
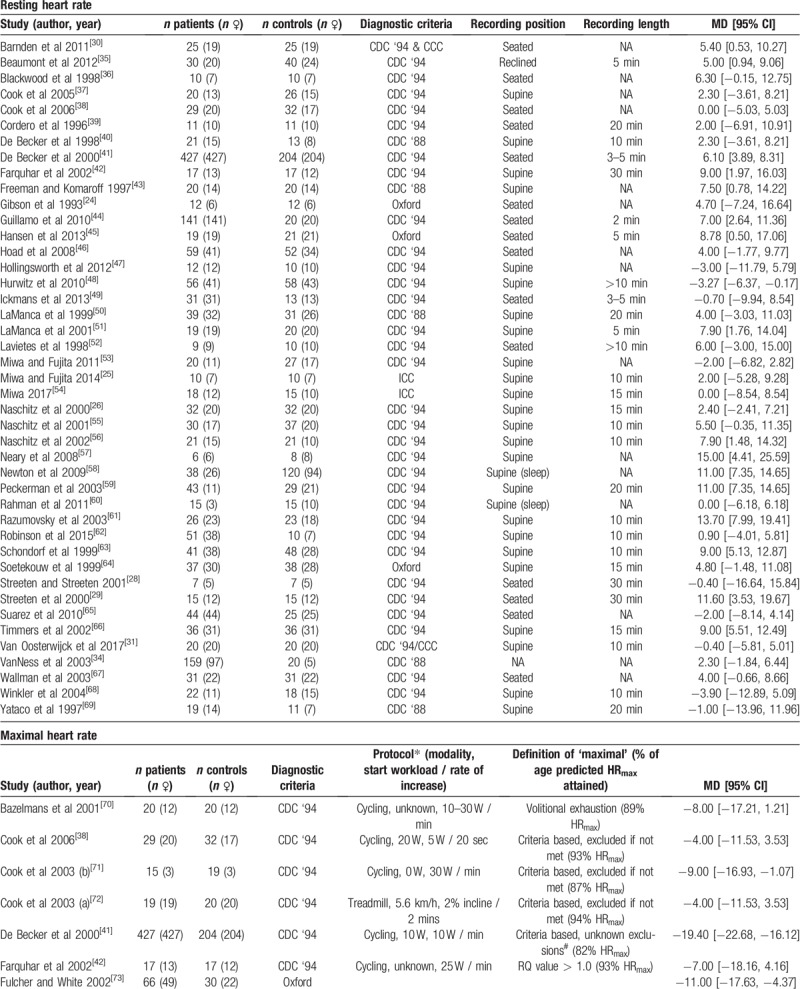
Summary of included studies, grouped by HR parameters assessed.

**Table 1 (Continued):**
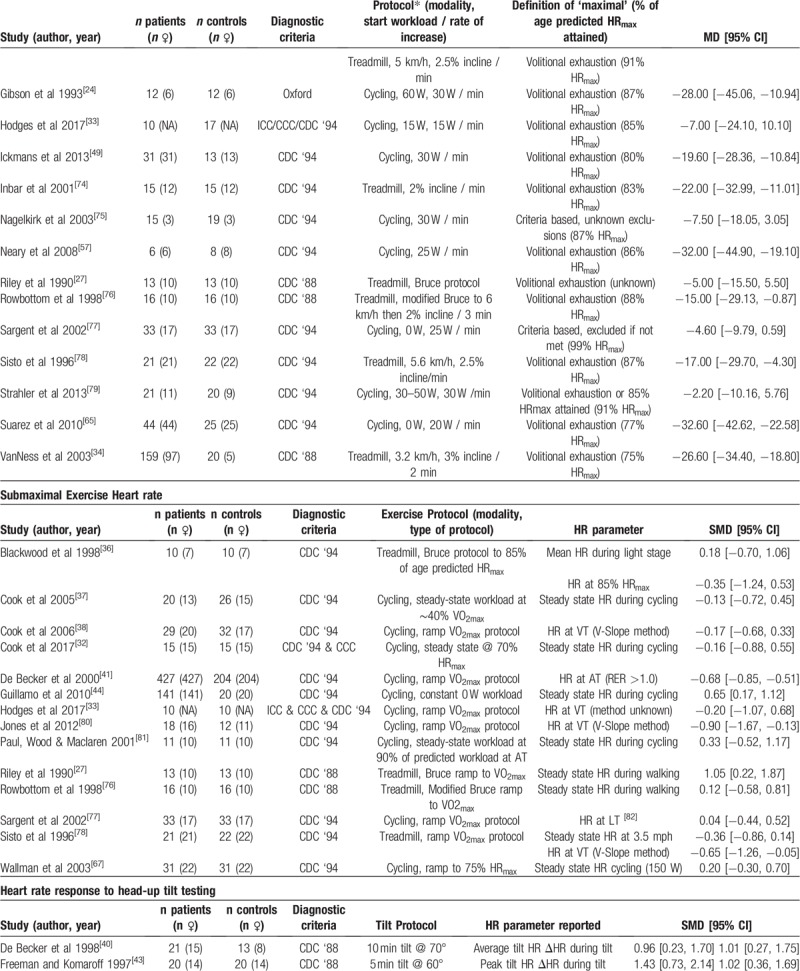
Summary of included studies, grouped by HR parameters assessed.

**Table 1 (Continued):**
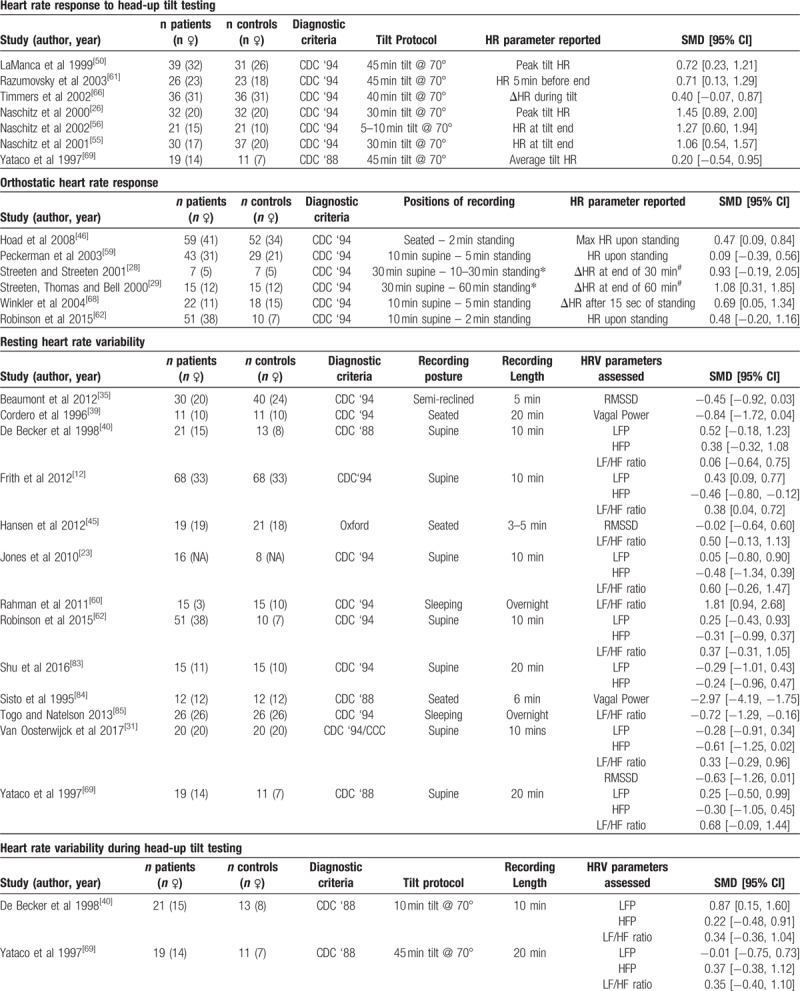
Summary of included studies, grouped by HR parameters assessed.

**Table 1 (Continued):**
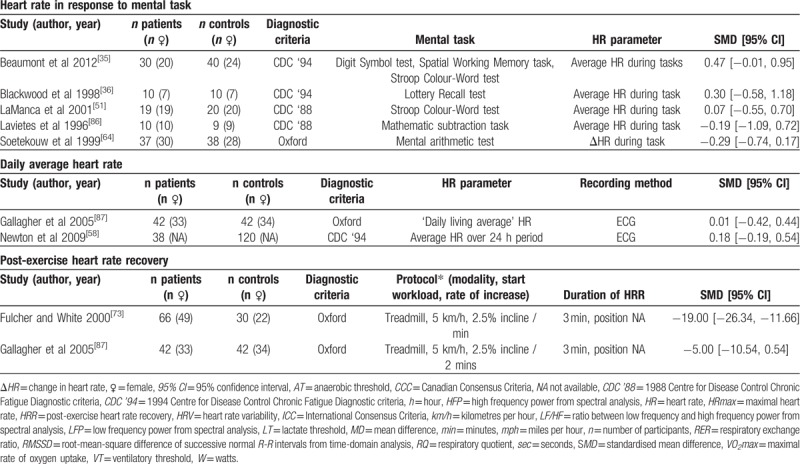
Summary of included studies, grouped by HR parameters assessed.

### 3.5 Resting heart rate

**Figure 2:**
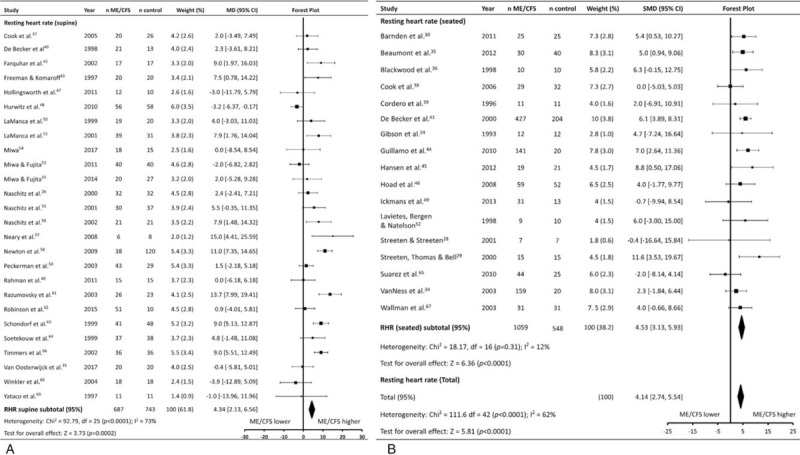
Effect of Myalgic Encephalomyelitis/Chronic Fatigue Syndrome on resting heart rate. Weight outside of parentheses indicates weighting within relevant subgroup analysis, weight inside parentheses indicates weighting within total resting heart rate analysis. CI = confidence interval, HR = heart rate, MD = mean difference, n number of participants, ME/CFS = myalgic encephalomyelitis/ chronic fatigue syndrome.

The most commonly assessed HR parameter was (RHR), which was assessed in 43 studies (patients n = 1766, controls n = 1291). Studies varied regarding the body position used to assess RHR, with 16 studies (patients n = 1059, controls n = 548) using a seated/reclined position (RHR_seated_), and 26 (patients n = 707, controls n = 743) using a supine position (RHR_supine_). Meta-analysis revealed ME/CFS patients had a RHR that was ∼4 beats/minute faster compared to controls (MD ± 95% CI = 4.14 ± 1.30, *P* < .001; Fig. 2), but with significant heterogeneity between studies (*P* < .001, *I*^2^ = 62%). Subgroup analysis revealed a similar difference between ME/CFS patients and controls for RHR_supine_ (4.02 ± 2.11, *P* < .001), and RHR_seated_ (4.53 ± 2.40, *P* < .001), with significant heterogeneity amongst studies evaluating RHR_supine_ (*P* < .001, *I*^2^ = 73%).

### 3.6 Maximal heart rate

HR_max_ was reported in 20 studies (patients n = 989, controls n = 568), which used 2 different exercise modalities (cycling, 13 studies; and treadmill walking, 7 studies) to elicit a maximal response. Five studies (patients n = 111, controls n = 123) – four of which employed a cycling protocol – based the attainment of a maximal response on accepted criteria and assessed true HR_max_, while the remaining 15 studies (patients n = 878, controls n = 445) only reported the highest HR achieved during testing, and is likely to represent symptom limited peak HR (HR_peak_), rather than a true HR_max_.

**Figure 3:**
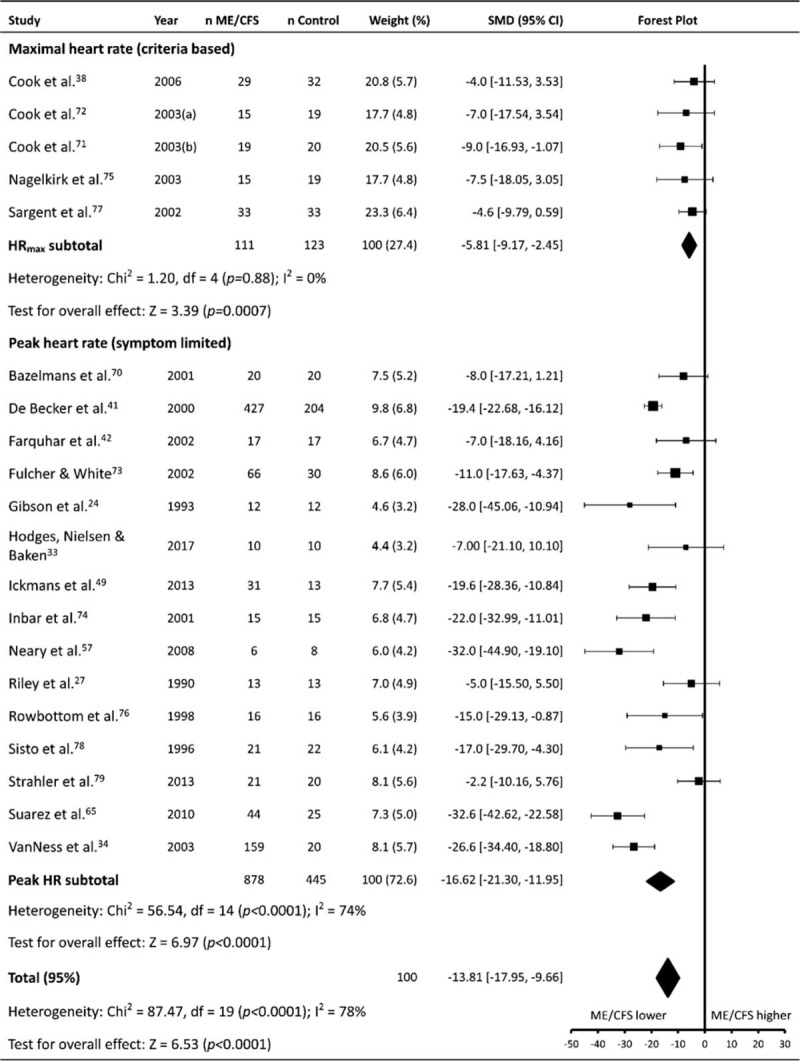
Effect of Myalgic Encephalomyelitis/Chronic Fatigue Syndrome on maximal heart rate. Weight outside of parentheses indicates weighting within relevant subgroup analysis, weight inside parentheses indicates weighting within total maximal heart rate analysis. CI = confidence interval, HR = heart rate, HRmax = maximal heart rate, MD = mean difference, ME/CFS = myalgic encephalomyelitis/chronic fatigue syndrome, n = number of participants.

Meta-analysis showed HR_max_ values were found to be lower for ME/CFS patients compared to controls (–13.81 ± 4.14, *P* < .001, Fig. 3), but were also affected by significant heterogeneity (*P* < .001, *I*^2^ = 78%). Subgroup analysis showed the difference between ME/CFS patients and controls was considerable in studies which measured symptom limited HR_peak_ (–16.62 ± 4.68, *P* < .001), with significant heterogeneity between studies (*P* < .001, *I*^2^ = 74%). Comparatively, in studies which assessed true HR_max_ the difference between ME/CFS patients and controls was smaller, albeit still significant (–5.81 ± 3.34, *P* < .001), with no heterogeneity present (*P* = .88, *I*^2^ = 0%).

### 3.7 Heart rate during submaximal exercise

HR during submaximal exercise was reported by 14 studies (patients n = 837, controls n = 496), with 10 studies using cycling exercise, and 4 studies using treadmill walking exercise (Table 1   ). Six studies (patients n = 539, controls n = 313) reported HR at a particular submaximal exercise ‘threshold’ (anaerobic threshold [AT], ventilatory threshold [VT], or lactate threshold [LT]), which were representative of the transition from predominately aerobic to predominately anaerobic energy systems (HR_threshold_). In addition, nine studies reported a steady state HR response (HR_steadystate_) at a given submaximal workload, with Blackwood et al^[36]^ reporting HR's at both a light absolute workload, and at a workload designed to elicit 85% of maximal HR. Seven (patients n = 244, controls n = 129) studies reported HR_steadystate_ during workloads that were likely below submaximal exercise threshold (AT, VT or LT), while 3 studies^[27,36,67]^ (patients n = 54, controls n = 54) reported a HR_steadystate_ that was likely to be at or above submaximal exercise threshold (AT, VT, or LT).

**Figure 4:**
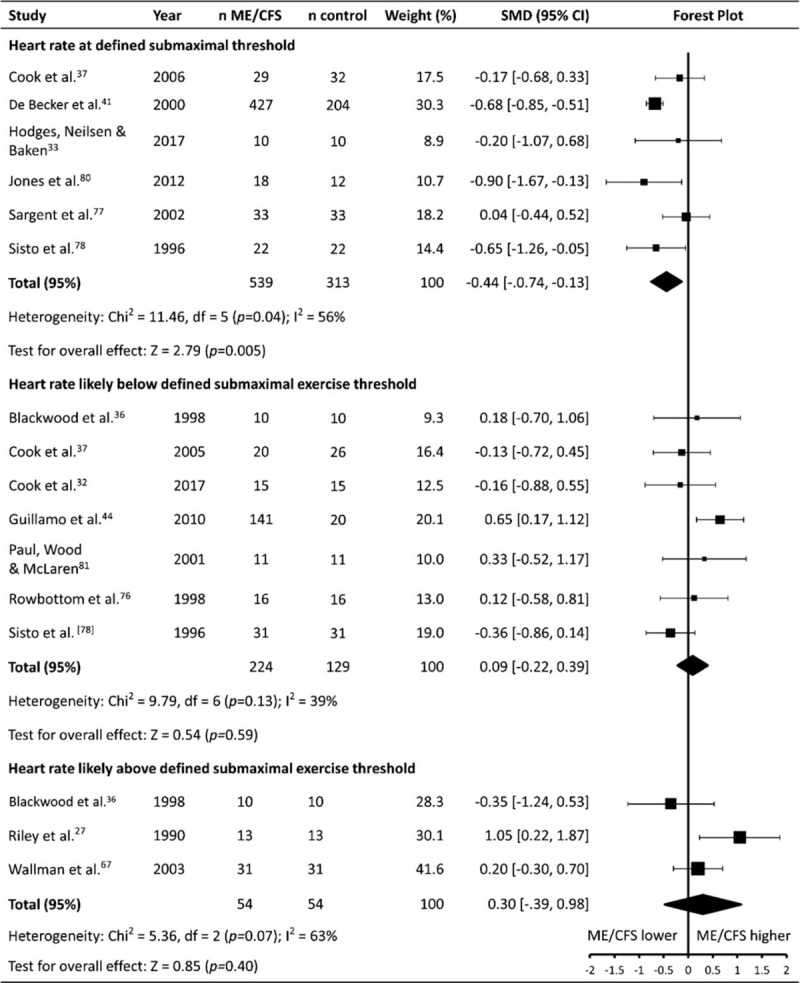
Effect of Myalgic Encephalomyelitis/Chronic Fatigue Syndrome on submaximal exercise heart rate. CI = confidence interval, HR = heart rate, ME/CFS = myalgic encephalomyelitis/chronic fatigue syndrome, n = number of participants, SMD = standardized mean difference.

Given the variations in the ways exercise HR was recorded and reported, with different thresholds being assessed in addition to varying exercise intensities at, above or below those thresholds, meta-analysis was performed separately for each HR parameter assessed during submaximal exercise. Analysis of HR_threshold_ revealed a moderate difference, with HRs being lower in ME/CFS patients compared to healthy controls (SMD ± 95% CI = –0.44 ± 0.31, *P* = .005), albeit with significant heterogeneity (*P* = .04, *I*^2^ = 56%; Fig. 4). No difference was found between ME/CFS patients and controls for HR_steadystate_ for workloads that were either likely below (0.09 ± 0.30, *P* = .59) or likely above submaximal exercise threshold (AT, VT, or LT) (0.30 ± 0.69, *P* = .40).

### 3.8 Heart rate during head-up tilt testing

**Figure 5:**
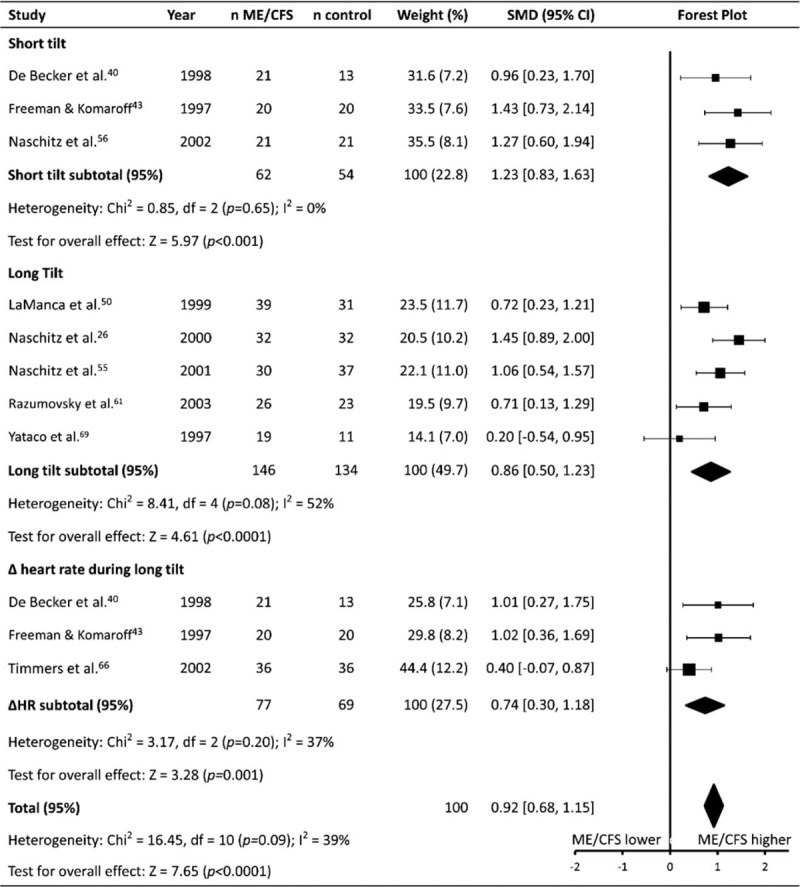
Effect of Myalgic Encephalomyelitis/Chronic Fatigue Syndrome on heart rate in response to head-up tilt testing. Weight outside of parentheses indicates weighting within relevant subgroup analysis, weight inside parentheses indicates weighting within total heart rate during HUTT analysis. CI = confidence interval, HR = heart rate, ME/CFS = myalgic encephalomyelitis/chronic fatigue syndrome, n = number of participants, SMD = standardized mean difference.

Nine studies (patients n = 285, controls n = 257) reported HR in response to HUTT (HR_tilt_) (Table 1   ). Eight studies (patients n = 208, controls n = 188) reported an absolute HR value during HUTT (peak HR, end HR, etc) and 3 studies (patients n = 77, controls n = 69) reported the ΔHR in response to HUTT.^[41,43,66]^ Meta-analysis revealed HR_tilt_ was higher in ME/CFS patients than it was in controls (SMD ± 95% CI = 0.92 ± 0.24, *P* < .001; Fig. 5). Subgroup analysis revealed that HR_tilt_ was increased in ME/CFS patients compared to controls, regardless of whether data were reported as the peak HR during a short (1.23 ± 0.40, *P* < .001) or long (0.86 ± 0.36, *P* < .001) duration HUTT, or if data were reported as the ΔHR during HUTT (0.74 ± 0.44, *P* = .001).

### 3.9 Orthostatic heart rate response

**Figure 6:**
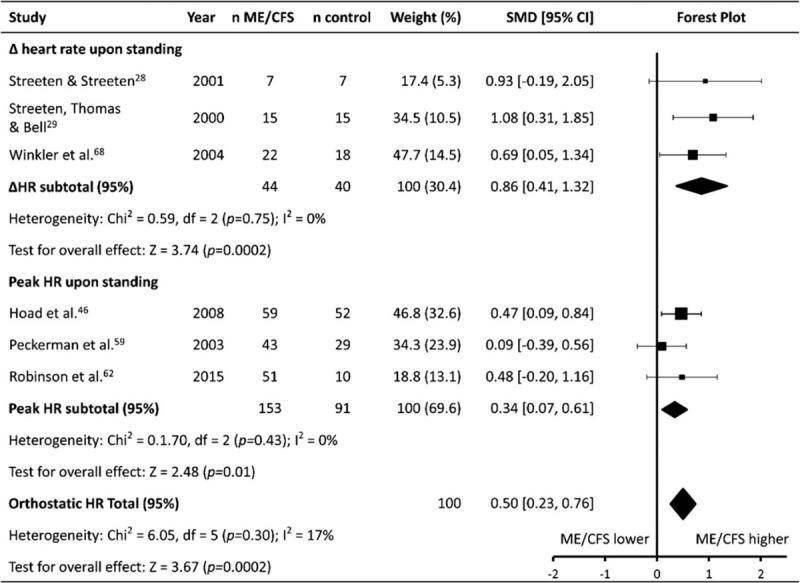
Effect of Myalgic Encephalomyelitis/Chronic Fatigue Syndrome on orthostatic heart rate response. Weight outside of parentheses indicates weighting within relevant subgroup analysis, weight inside parentheses indicates weighting within total orthostatic response heart rate analysis. CI = confidence interval, HR = heart rate, ME/CFS = Myalgic Encephalomyelitis/Chronic Fatigue Syndrome, n = number, SMD = standardized mean difference.

The orthostatic HR response (HR_OR_) was reported in 6 studies (patients n = 197, controls n = 131) (Table 1   ). Five studies^[28,29,59,62,68]^ reported HR_OR_ in response to standing up after a period of supine rest, while 1 study^[46]^ reported the effect of standing up following sitting. Meta-analysis showed HR_OR_ was higher for ME/CFS patients compared to controls (SMD ± 95% CI = 0.50 ± 0.27, *P* < .001; Fig. 6). Subgroup analysis revealed there was a moderate difference when HR_OR_ was reported as the ΔHR upon moving from lying/sitting to standing, with ME/CFS patients having a higher ΔHR than controls (0.86 ± 0.46, *P* < .001), with a similar finding, albeit of a smaller magnitude, also found for the peak HR obtained upon standing (0.34 ± 0.27, *P* = .01).

### 3.10 Resting heart rate variability

Measures of resting HRV (HRV_rest_) were reported in 13 studies (patients n = 294, controls n = 270) (Table 1   ). Seven studies recorded HRV_rest_ in a supine position, while 3 ^[39,45,84]^ recorded HRV_rest_ while seated/reclined. Two studies^[60,85]^ recorded HRV_rest_ during overnight sleep. Frequency domain HRV_rest_ measures were used in 12 studies, with 1 study^[35]^ reporting only time domain analyses, and 2 studies^[31,45]^ reporting both frequency and time domain analyses.

**Figure 7:**
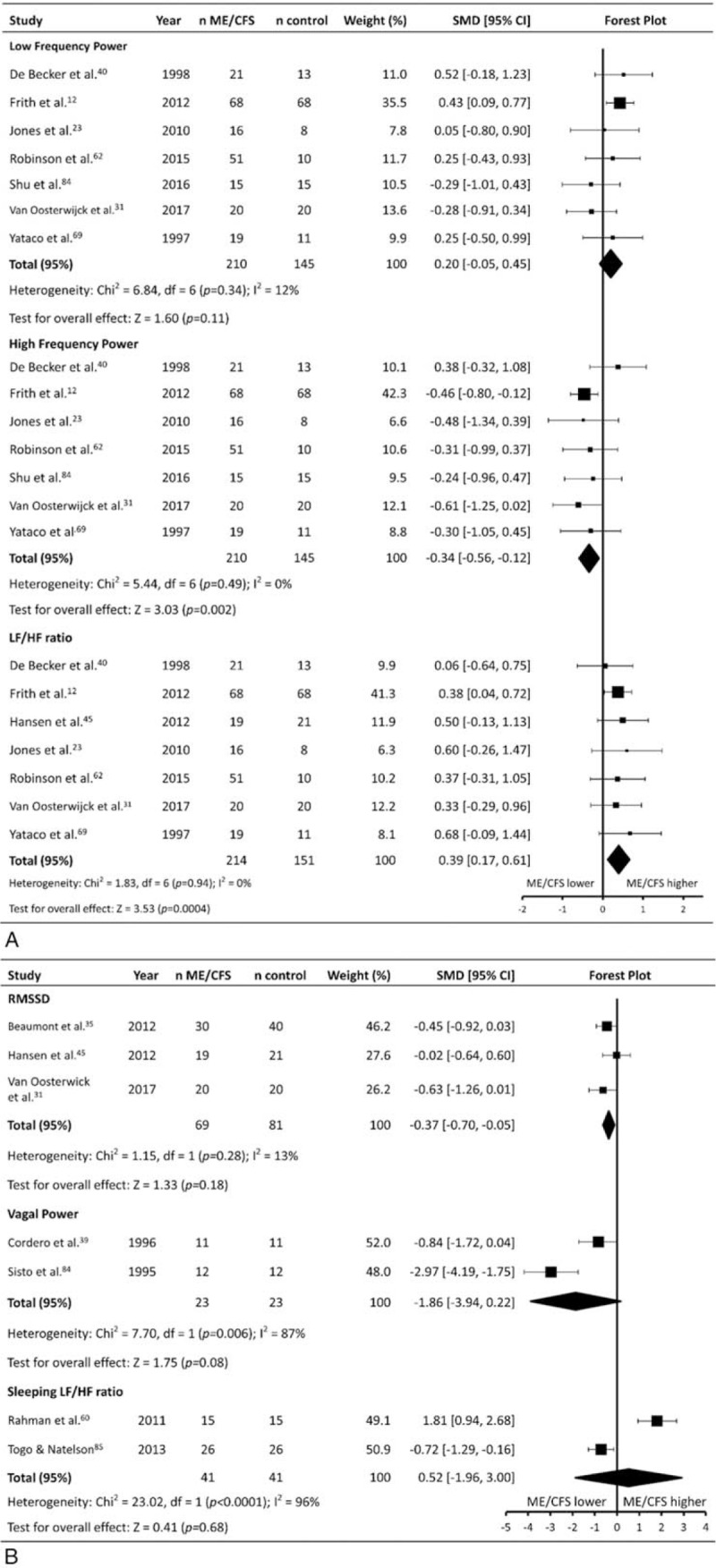
Effect of Myalgic Encephalomyelitis/Chronic Fatigue Syndrome on resting heart rate variability. CI = confidence interval, LF/HF = low frequency power/high frequency power, ME/CFS = Myalgic Encephalomyelitis/Chronic Fatigue Syndrome, n = number of participants, RMSSD = root-mean-square difference of successive normal R-R intervals from time-domain analysis, SMD = standardized mean difference.

Parameters of HRV_rest_ from included studies were meta-analysed separately. ME/CFS patients had a higher ratio between low frequency and high frequency power (LF/HF) (SMD ± 95% CI = 0.20 ± 0.25, *P* = .11) than controls, and a lower high frequency power (HFP) (–0.34 ± 0.22, *P* = .002) and root mean of the sum of squares of beat to beat deviations (RMSSD) (–0.37 ± 0.32, *P* *=* .02) (Fig. 7). No differences were found for low frequency power (LFP) (0.39 ± 0.22, *P* < .001), Vagal Power (–1.86 ± 2.08, *P* = .08), or sleeping LF/HF ratio (–0.52 ± 2.48, *P* *=* .68), with significant heterogeneity for Vagal Power (*P* = .006, *I*^2^ = 87%), and sleeping LF/HF (*P* < .001, *I*^2^ = 96%).

### 3.11 Heart rate variability during head-up tilt testing

**Figure 8:**
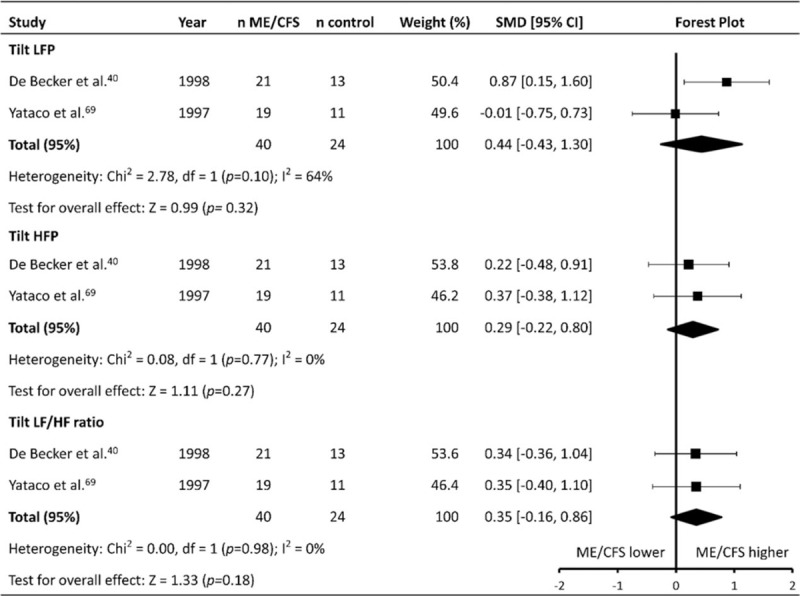
Effect of Myalgic Encephalomyelitis/Chronic Fatigue Syndrome on heart rate variability during head-up tilt testing. CI = confidence interval, HFP = high frequency power, LF/HF = low frequency/high frequency, LFP = Low frequency power, ME/CFS = Myalgic Encephalomyelitis/Chronic Fatigue Syndrome, n = number of participants, SMD = standardized mean difference.

Two studies (patients n = 40, controls n = 24) reported HRV during HUTT^[40,69]^ (HRV_tilt_) (Table 1   ). Both studies reported exclusively frequency domain parameters, with each reporting on LFP, HFP, and LF/HF ratio. Meta-analysis found no difference between ME/CFS patients and controls for LFP (SMD ± 95% CI = 0.44 ± 0.87, *P* = .32; Fig. 8), HF power (0.29 ± 0.51, *P* = .27), or LF/HF ratio (0.35 ± 0.51, *P* = .18).

### 3.12 Heart rate during mental task(s)

**Figure 9:**
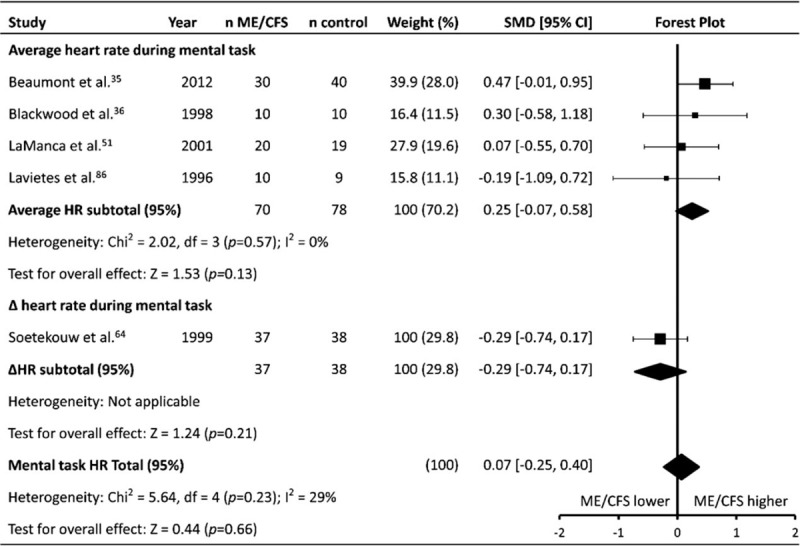
Effect of Myalgic Encephalomyelitis/Chronic Fatigue Syndrome on heart rate response to mental task. Weight outside of parentheses indicates weighting within relevant subgroup analysis, weight inside parentheses indicates weighting within total mental task heart rate analysis. CI = confidence interval, HR = heart rate, ME/CFS = Myalgic Encephalomyelitis/Chronic Fatigue Syndrome, n = number, SMD = standardized mean difference.

Five studies (patients n = 107, controls n = 116) reported the HR response to a mental task (HR_mentaltask_) (Table 1   ). Subgroup analysis revealed no significant effect for HR_mentaltask_ when reported as the average HR during the tasks in 4 studies^[35]^^ 6451,^^[86]^ (SMD ± 95% CI = 0.25 ± 0.32, *P* = .57) (Fig. 9). One study reported HR_mentaltask_ as the ΔHR during task, again with no significant difference between ME/CFS and controls (-0.29 ± 0.45, *P* = .21).

### 3.13 Daily average heart rate

**Figure 10:**
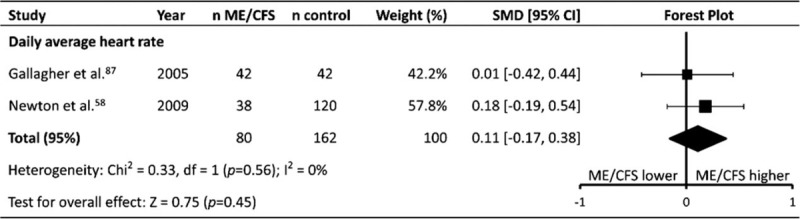
Effect of Myalgic Encephalomyelitis/Chronic Fatigue Syndrome on daily average heart rate. CI = confidence interval, ME/CFS = Myalgic Encephalomyelitis/Chronic Fatigue Syndrome, n = number of participants, SMD = standardized mean difference.

Daily average HR (HR_dailyaverage_) was reported in 2 studies^[58,87]^ (patients n = 80, controls n = 162) (Table 1   ). Both studies used the same measurement techniques and reported data in the same way – HR data were measured through ECG monitoring, and both studies reported the average HR over 24 hours. Meta-analysis revealed no difference between the ME/CFS patients and controls (SMD ± 95% CI = 0.11 ± 0.27, *P* = .45; Fig. 10), with no heterogeneity between the studies (*P* = .56, *I*^2^ = 0%).

### 3.14 Post-exertional heart rate recovery

**Figure 11:**
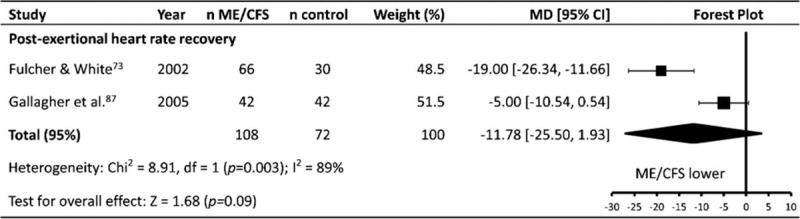
Effect of Myalgic Encephalomyelitis/Chronic Fatigue Syndrome on post-exercise heart rate recovery. CI = confidence interval, MD = mean difference, ME/CFS = Myalgic Encephalomyelitis/ Chronic Fatigue Syndrome, n = number of participants.

HRR was reported in only 2 articles (patients n = 108, controls n = 72), both of which used similar methods to elicit and record HRR (Table 1   ). Meta-analysis found no difference in HRR between ME/CFS patients and controls (MD ± 95% CI = –11.78 ± 13.72, *P* = .09; Fig. 11), with results affected by significant statistical heterogeneity (*P* = .003, *I*^2^ = 89%).

## 4 Discussion

This review found a number of differences in HR parameters between patients with ME/CFS and controls. Meta-analysis identified that compared to controls, patients with ME/CFS exhibited higher resting HR (RHR), lower maximal/peak HR (HR_max_/HR_peak)_, higher HR responses to HUTT (HR_tilt_) and moving from sitting to standing (HR_OR_), and lower HR at submaximal exercise threshold (HR_threshold_). Resting HRV (HRV_rest_) parameters also differed between ME/CFS patients and controls, with patients exhibiting higher LFP and lower HFP. Taken together, these results tend to suggest reduced vagal modulation and increased sympathetic modulation of HR in patients with ME/CFS. Included studies were generally of a moderate to good quality, and although confounding factors were rarely identified and dealt with it is unlikely that the quality of included articles played a major role on the results of this meta-analysis.

### 4.1 Resting heart rate (RHR)

RHR was higher for ME/CFS patients compared to controls, regardless of whether assessed when supine or seated. The higher RHR likely reflects a decrease in parasympathetic and increase in sympathetic cardiac modulation as multiple studies have shown that both parasympathetic blockade and an increase in sympathetic autonomic control result in an increase in RHR.^[88,89]^ Interestingly, the analysis of RHR_supine_ was affected by significant heterogeneity, which was not the case for RHR_seated_. Very few studies made any effort to control breathing rate in either body position, and it is likely that the potential difference in breathing rates between studies may account for some of the variation in RHR values reported.^[90]^ Although RHR appears to be higher for patients with ME/CFS than controls, the average difference between groups (4.33 ± 1.42 bpm) is less than the typical day to day variation of RHR reported in the literature (∼5 bpm).^[91]^ Therefore, while the increased RHR experienced by ME/CFS patients may indicate an altered autonomic balance, it may not be a clinically relevant finding as the large amount of day to day variation in RHR will limit its potential utility as a diagnostic marker in ME/CFS. It must be acknowledged that there was a large amount of variability in the methods utilised to capture RHR data in the included studies including the duration of rest utilised, with rest periods ranging from ≤ 5 minutes to 30 minutes, while 15 studies did not report the duration of rest utilised.

### 4.2 Heart rate variability (HRV_rest_ and HRV_tilt_)

In addition to ME/CFS patients having a higher RHR, they exhibited differences in HRV_rest_ that also suggest the presence of reduced parasympathetic and increased sympathetic cardiac autonomic modulation. ME/CFS patients had lower HFP and RMSSD, with both parameters representing primarily parasympathetic cardiac modulation.^[92,93]^ ME/CFS patients also had a higher LF/HF ratio than controls, indicating increased sympathetic and decreased parasympathetic HR modulation compared to controls.^[94]^ The effect sizes for the differences between ME/CFS patients and controls were consistently small (albeit significant), and this may explain why no significant effect was found for other HRV_rest_ parameters which had fewer included studies. LFP, for example, reflects a combination of parasympathetic and sympathetic modulation of HR vagal cardiac modulation in a similar manner to HFP, however the small number of included studies for this parameter may limited the ability for the current meta-analysis to identify a significant effect. Although it is important to note that the effect sizes seen for the HRV_rest_ analyses were small, and there were noticeably fewer included studies than for the analysis of parameters such as RHR, the consistent effect on HRV parameters (RMSSD HFP, LF/HF ratio) suggests that HRV_rest_ may be a useful marker of autonomic dysfunction in ME/CFS patients to assist with the diagnosis of the condition. Interestingly, analysis of HRV_tilt_ found no difference in any parameter between ME/CFS patients and controls, indicating there is no difference in HRV between the groups when an autonomic stressor is introduced, although again, this may be due to the small number of included studies (n = 2).

### 4.3 Maximal heart rate (HR_max_)

Meta-analysis also revealed a lower HR_max_ in ME/CFS patients compared to controls, which may represent further evidence of increased sympathetic modulation of HR. Lower HR_max_ was found between ME/CFS patients and controls for studies which used set criteria to determine if a maximal effort had been produced and likely reported true HR_max_, as well as studies which did not use a criteria approach and likely reported symptom-limited peak HR (HR_peak_). Despite obvious differences arising from the 2 approaches, the fact that HR_max_ was lower in ME/CFS patients in studies when criterion measures were applied suggests that there is a lowering of true HR_max_ in patients with ME/CFS compared with controls. When considered in the context of resting HRV measures indicating increased sympathetic and decreased parasympathetic tone, the lower HR_max_ in ME/CFS patients may be due to resting sympathetic hyperactivity. Alterations in blood pressure variability have also been found in ME/CFS patients^[12,95,96]^ which indicate increased sympathetic autonomic regulation, and it is possible that prolonged pathological sympathetic activity has led to ME/CFS patients’ autonomic effectors (such as heart and blood vessels) becoming resistant to sympathetic autonomic stimulation through downregulation of receptors,^[12]^ thus contributing to the lower HR_max_ identified in this review. Additionally, patients with congestive heart failure have been shown to exhibit abnormally high levels of sympathetic activity, combined with decreased cardiac norepinephrine, suggesting that periods of increased sympathetic neural tone may result in a depletion of cardiac sympathetic neurotransmitters.^[97]^ However, despite the clear differences in HR_max_/HR_peak_ between ME/CFS patients and controls, it is likely to have limited usefulness as a diagnostic marker in ME/CFS. It is notoriously difficult to elicit a reliable and valid maximal effort in clinical populations^[98]^ and the high intensity exercise required to elicit HR_max_ is likely to cause a significant exacerbation of symptoms for ME/CFS patients.^[99]^ Additionally, the wide variability in individual HR_max_ values (standard deviation ∼ 10 bpm)^[100]^ suggests that, despite the apparent differences between patients and controls, the diagnostic accuracy at an individual level would be low.

The assessment of peak HR_max_/HR_peak_ in the included studies was affected by numerous methodological issues which impacted on the consistency of results seen during studies which assessed symptom limited HR_peak_, rather than true HR_max_. The effect on true HR_max_ was very consistent, with minimal heterogeneity (*P* = .88, *I*^2^ = 0%), compared to those which measured HR_peak_ where the heterogeneity between studies was much larger (*P* < .0001, *I*^2^ = 74%). The heterogeneity in the studies which used reported HR_peak_ is likely, in part, due to differences in encouragement given to participants during the maximal exercise testing. Some studies^[78,79]^ reported that no encouragement was given during testing, while others^[42,70]^ reported that verbal encouragement was given in an attempt to elicit a maximal response. Regular verbal encouragement during maximal exercise testing has been shown to elicit a greater maximum effort compared to when no encouragement is given.^[101]^ As ME/CFS patients (similarly to most chronic health conditions) have been shown to catastrophize their symptoms of pain, leading to a negative effect on exercise performance,^[102]^ the use of regular verbal encouragement during maximal exercise tests in ME/CFS may be particularly important in order to elicit a valid maximal response in this population. The ability for the included studies to elicit a valid maximal response in the patient group can be established from analysis of the percentage of age predicted HR_max_ achieved during data collection. Across the studies which reported HR_peak_/true HR_max_, the percentage of age predicted HR_max_ obtained in each study varied from 99% in the study by Sargent et al^[77]^ which relied on a criterion approach, to 75% in a study by VanNess et al^[34]^ which reported symptom limited HR_peak_. One study^[27]^ did not report the age of the participants, so predicted maximal HR was unable to be calculated. The 3 highest % age predicted HR_max_ attained by studies were all those which employed a criterion based approach to determine if a maximal effort was given^[38,72,77]^ suggesting this approach may result in better assessment of HR_max_ in this clinical population

### 4.4 Heart rate response to head-up tilt testing (HR_tilt_)

In addition to the evidence of sympathetic overactivity in ME/CFS patients provided by analysis of RHR, HRV_rest_, and HR_max_, the results from the analysis of HR_tilt_ further suggest an altered autonomic balance in ME/CFS patients compared to controls, revealing that ME/CFS patients had a significantly higher HR during HUTT, in addition to a higher ΔHR in response to HUTT. This difference in HR_tilt_ is potentially due to an increased sympathetic tone which may be exhibited ME/CFS patients.^[43]^ Additionally, Timmers et al^[66]^ found a blunted plasma norepinephrine response to HUTT in patients who experienced presyncope without preceding tachycardia, further indicating the presence of altered autonomic function during HUTT in ME/CFS patients. However, it must be acknowledged that variability existed in the methodology utilised to collect HR_tilt_ data. While studies were generally consistent in the tilt methodology used, (all studies using a 70° tilt, with the exception of Freeman and Komaroff^[43]^ who used a 60° tilt) thre was large variability in the durations of tilt employed: 3 studies utilised durations of ≤10 minutes,^[40,43,56]^ while the remainder used durations of 30 to 40 minutes.

### 4.5 Orthostatic heart rate response (HR_OR_)

Similar to the findings for HR_tilt_, ME/CFS patients were also found to have an increased HR_OR_ compared to controls. This finding is not surprising given the well-known co-morbidities of ME/CFS and postural orthostatic tachycardia syndrome (POTS). Multiple studies have found that rates of POTS are significantly higher in ME/CFS populations,^[103,104]^ and the greater HR_OR_ seen in ME/CFS participants may be due to an increased sympathetic/parasympathetic balance.^[68,105]^ Similar to HR_tilt_ however, significant variability existed in the methodologies utilised to collect HR_OR_ data which limits the ability to draw definite conclusions about the patterns observed. For example, 5 studies^[28,29,59,62,68]^ reported HR_OR_ in response to standing up after a period of supine rest, while 1 study^[46]^ reported the effect of standing up following sitting, with durations spent in the resting position prior to standing also varying across studies (from 10 minutes^[68]^ to 30 minutes^[28]^. Further, durations of standing differed greatly, ranging from short (2 minutes^[46,62]^) to long (60 minutes^[29]^).

Although the results of the meta-analysis suggest that ME/CFS patients have an altered autonomic balance compared to controls – consisting of higher resting sympathetic and lower resting parasympathetic cardiac modulation – it could be argued that these findings reflect the physical deconditioning that has been found in some ME/CFS patients.^[106]^ Due to the exacerbation of post-exertional malaise which commonly occurs as a result of physical activity in this population, ME/CFS patients are candidates for physical deconditioning as a consequence of exercise avoidance.^[102,107]^ For example, physical deconditioning is a potential cause of the increased RHR experienced by ME/CFS patients. Previous work by De Lorenzo et al,^[106]^ which was subsequently confirmed by Miwa and Fujita,^[108]^ found that ME/CFS patients had a reduced left ventricular size and mass, and such reductions in left ventricular size and mass are typically associated with deconditioning in healthy individuals.^[109]^ Additionally, multiple studies have shown that a decreased level of aerobic fitness is associated with a lower vagal and increased sympathetic tone which can be reversed through aerobic training,^[110,111]^ implying the altered HRV_rest_ found in ME/CFS patients may result from the deconditioning typical of ME/CFS, rather than being a direct cause or symptom of the syndrome. Further, the elevated HR_tilt_ for ME/CFS patients may also potentially be due to physical deconditioning. LaManca et al^[50]^ measured stroke volume during HUTT in ME/CFS patients, and found stroke volume decreased in a similar manner to that seen in other deconditioned populations,^[112]^ and suggested the increased HR_tilt_ witnessed during that study attempted to counteract a hypovolaemic state which had led to decreased preload and decreased stroke volume. Similar observations have been made by other studies in ME/CFS populations.^[43,66]^

### 4.6 HR during submaximal exercise

The finding of lower HRs at submaximal exercise threshold, such as AT, LT, or VT (HR_threshold_) in ME/CFS participants compared to controls may be further indication of potential physical deconditioning, given that it is well established that an increased level of aerobic fitness is associated with a later onset of submaximal exercise threshold.^[113–115]^ Interestingly, in 4 of the 5 studies which reported on HR at VT/AT,^[38,41,78,80]^ ME/CFS participants had a lower peak VO_2_ than controls, providing further evidence of deconditioning while the fifth study^[33]^ was underpowered to find a difference between the 2 groups for this parameter. It is important to note that the analysis of HR_threshold_ was affected by significant, moderate heterogeneity (*I*^2^ = 56%), which likely resulted from the different methods used to quantify submaximal exercise threshold. Three of the studies^[38,78,80]^ used the V-Slope method to determine VT, while De Becker et al^[41]^ used an RER < 1.0 to determine that AT had been reached, and Sargent et al^[77]^ calculated lactate threshold using the method of Beaver et al.^[82]^ Hodges et al^[33]^ reported on HR at VT, but did not state the method used to quantify VT. Indeed there remains debate as to whether or not there is a relationship between AT, LT, and VT, with some^[114]^ arguing for a strong physiological link between the thresholds, and others believing the similar timing of the 2 thresholds to be purely coincidental.^[116]^ The study by Sargent et al^[77]^ which assessed HR at LT found no difference between ME/CFS patients and controls. Comparatively, 3 other studies which assessed HR at VT/AT found that HR was lower for patients than controls.^[41,78,80]^ While this suggests plasma lactate measures may differ from ventilatory measures in their ability to reflect differences between ME/CFS patients and controls, the differences in VO_2max_ in the studies which assessed HR at AT/VT suggest these differences may also result from physical deconditioning in the ME/CFS patients. Interestingly, there was no difference in the HR_steadystate_ of ME/CFS patients and controls. It is well known that HR_steadystate_ in response to equal workload is generally higher in individuals with a lower level of aerobic fitness^[117,118]^ and 2 of the 3 studies which reported both HR_steadystate_ and VO_2max_^[27,78]^ found lower VO_2max_ values for ME/CFS patients when compared to healthy controls. Therefore, it is surprising that there was no difference between the HR_steadystate_ values reported between ME/CFS patients and controls in response to an equal workload.

### 4.7 Other heart rate parameters (HR_dailyaverage_, HR_mentaltask_, HRR)

No difference was found between ME/CFS patients and controls for HR_dailyaverage_, HR_mentaltask_, or HRR, potentially due to the small number of included studies for each parameter. HR_mentaltask_ was reported in 5 studies, while HR_dailyaverage_^[58,87]^ and HRR^[73,87]^ were both reported in 2 studies. Given that ME/CFS patients had a higher RHR than controls, it may be expected that a similar difference would be apparent during tasks which do not require physical exertion, such as a mental task. Similarly, it may be expected that ME/CFS patients would have a higher HR_dailyaverage_ compared to controls due to a combination of physical deconditioning and an apparent sympathetic dominance at rest, however this does not appear to be the case. With regard to HR_dailyaverage,_ this may be due to increased patterns of exercise avoidance that ME/CFS patients exhibit^[107,119]^ leading to lower daily physical activity,^[120]^ and therefore an overall similar HR_dailyaverage_ compared to controls. No difference was found between ME/CFS patients and controls with regard to post exercise HRR. The analysis only included 2 studies,^[73,87]^ and was affected by significant statistical heterogeneity (*P* = .003, *I*^2^ = 89%), likely due to the small number of included studies. One study^[73]^ found a 19 bpm difference between ME/CFS patients and controls, and the other^[87]^ found no difference. Regardless, HRR is unlikely to be a suitable marker for use in an ME/CFS patient group, not only because of the lack of current evidence indicating any difference between patients and controls, but also because the assessment of HRR has been found to require exercise intensities of 88% of maximal HR in order to minimise day-to-day variation in athletes.^[121]^ Given the difficulties that many ME/CFS patients may have in attempting to attain this elevated HR, the reliability of HRR may therefore be diminished in ME/CFS patients, in addition to the fact that high intensity exercise can exacerbate ME/CFS symptoms.^[122]^

Interestingly, of the studies which reported maximal HR in ME/CFS patients, the majority that also reported VO_2max_ (10 of 17) found a lower VO_2max_ in ME/CFS patients compared to controls, which could suggest that deconditioning is common in ME/CFS. However, of these studies, four of them were those which used a criterion approach to determine if a valid maximal effort had been given, and these studies found no difference in VO_2max_ between ME/CFS and controls, implying that many ME/CFS patients may not be deconditioned compared to controls, but are unable to produce a valid voluntary maximal effort, possibly due to kinesiophobia.^[102,107]^ While some studies have found differences in aerobic fitness between ME/CFS patients and controls, and it is possible that deconditioning may be a potential explanation for some of the differences in HR parameters seen between ME/CFS patients and controls, the equivocal findings regarding aerobic fitness parameters (with some studies reporting a difference between ME/CFS and controls,^[42,74]^ and others not^[38,75]^) suggest that deconditioning cannot explain all of the variance in HR parameters between ME/CFS patients and controls. Rather, it is likely that an altered cardiac autonomic balance is present in ME/CFS patients, typified by increased sympathetic and decreased parasympathetic cardiac autonomic control. In any case, while deconditioning through avoidance of physical activity may have had an effect on the HR analyses in this meta-analysis, it is important to note that physical deconditioning is not a potential cause of ME/CFS, but rather it occurs as a consequence due to symptoms (i.e. fatigue, pain) reducing the capacity for participation in physical activity which may be further compounded by active avoidance of activity even when able, for fear of inducing post-exertional malaise and severe exacerbation of symptoms.^[102]^

## 5 Limitations

This review and meta-analysis included ME/CFS patients who were diagnosed with any of a number of recognised criteria. Although the 1994 CDC criteria^[1]^ was by far the most commonly used criteria in the included studies, it may not be the most effective tool for clinical diagnoses, a function for which it was never intended.^[3]^ In recent times, the ‘Canadian Criteria’ (CCC)^[3]^ and the International Consensus Criteria on ME/CFS (ICC)^[4]^ have been preferred by many clinicians as they require the patient to experience an acute worsening of symptoms with exercise, something which was not required as part of the 1988 or 1994 CDC definitions. However, very few of the articles included in this review employed the CCC or the ICC as their diagnostic criteria. Given the differences between the diagnostic criteria, it cannot be discounted that there are differences in the characteristics of the illnesses between studies which used different diagnostic criteria. Future research in ME/CFS populations should ideally report the number of included participants who met the 1994 CDC criteria (made to standardise research), and what number met the ICC or CCC (made for better clinical diagnosis).

Where possible, this review attempted to explain the reasons for any statistical heterogeneity that was identified during the meta-analysis process. Although this heterogeneity may be the result of methodological issues, it is likely that the results for each study are affected by the disease severity of included patients, in addition to the duration of illness. While some included studies^[34,59]^ did classify patients into subcategories based on symptom severity, the majority of included studies did not. With particular regard to the HR parameters recorded during or following exercise (HR_max_, HRR, exercise HR), selection bias is a potential issue that may limit the validity of the results from included studies, as patients who were more severely affected would be less likely to volunteer for exercise-based studies. Additionally, given that ME/CFS patients may be affected by cardiovascular deconditioning as a result of their condition, conceivably, ME/CFS patients who have had the illness for a longer duration are likely to have experienced greater deconditioning, and this increased deconditioning may explain some of the statistical heterogeneity witnessed for some parameters. Additionally, it must be acknowledged that it was not possible to analyse results based on other factors (e.g., level of physical activity) due to the under-reporting of such details within included studies, or a tendency to report all results within a single sample despite differences in participant characteristics (e.g., male or female).

## 6 Conclusions

Numerous HR parameters have been reported on in ME/CFS patients, with wide variations in study design and data acquisition methods, including body position and the duration/intensity of interventions (HUTT, exercise etc). Meta-analysis revealed significant differences between patients and controls in a number of parameters, including patients having: higher RHR, HR_tilt_, orthostatic HR response, and LF/HF ratio; and lower HR_max_, HR_threshold_, HFP and RMSSD. These differences suggest an altered regulation of HR in ME/CFS patients that is suggestive of reduced vagal and increased sympathetic modulation of heart rate. It does not appear that any of the currently used HR parameters have the sensitivity to detect the presence of ME/CFS on their own, as demonstrated by the presence of high levels of statistical heterogeneity and methodological issues which limit the usefulness of these parameters. However, the results of this review suggest that there are quantifiable differences in autonomic HR regulation in ME/CFS patients, and future research in ME/CFS populations should therefore focus on determining if there are additional HR parameters which have diagnostic utility in this group.

## Author contributions

**Conceptualization:** Maximillian James Nelson, Jonathan D. Buckley, Rebecca L. Thomson, Kade Davison.

**Data curation:** Maximillian James Nelson, Jasvir S Bahl.

**Formal analysis:** Maximillian James Nelson, Jasvir S Bahl.

**Investigation:** Maximillian James Nelson, Jasvir S Bahl, Jonathan D. Buckley, Rebecca L. Thomson, Kade Davison.

**Methodology:** Maximillian James Nelson, Jonathan D. Buckley, Rebecca L. Thomson, Kade Davison.

**Project administration:** Jonathan D. Buckley.

**Supervision:** Jonathan D. Buckley, Rebecca L. Thomson, Kade Davison.

**Validation:** Maximillian James Nelson.

**Writing – original draft:** Maximillian James Nelson.

**Writing – review & editing:** Maximillian James Nelson, Jasvir S Bahl, Jonathan D. Buckley, Rebecca L. Thomson, Kade Davison.

Maximillian James Nelson orcid: 0000-0003-1759-1428.

## References

[1] Fukuda K Straus SE Hickie I The chronic fatigue syndrome: a comprehensive approach to its definition and study. Ann Intern Med 1994;121:953 9. 7978722 10.7326/0003-4819-121-12-199412150-00009

[2] Holmes GP Kaplan JE Gantz NM Chronic fatigue syndrome: a working case definition. Ann Intern Med 1988;108:387 9. 2829679 10.7326/0003-4819-108-3-387

[3] Carruthers BM Jain AK De Meirleir KL Myalgic Encephalomyelitis/Chronic Fatigue Syndrome: clinical working case definition,;1; diagnostic and treatment protocols. J Chron Fatigue Syndr 2003;11:7 115.

[4] Carruthers BM van de Sande MI De Meirleir KL Myalgic Encephalomyelitis: international consensus criteria. J Intern Med 2011;270:327 38. 21777306 10.1111/j.1365-2796.2011.02428.x PMC3427890

[5] Yiu Y Qiu M A preliminary epidemiological study and discussion on traditional Chinese medicine pathogenesis of chronic fatigue syndrome in Hong Kong. J Chin Integr Med 2005;3:359 62. 10.3736/jcim20050506 16159567

[6] Nacul LC Lacerda EM Pheby D Prevalence of myalgic encephalomyelitis/chronic fatigue syndrome (ME/CFS) in three regions of England: a repeated cross-sectional study in primary care. BMC Med 2011;9:1 2. 21794183 10.1186/1741-7015-9-91 PMC3170215

[7] Johnston S Brenu EW Staines D The prevalence of chronic fatigue syndrome/myalgic encephalomyelitis: a meta-analysis. Clin Epidemiol 2013;5:105 10. 23576883 10.2147/CLEP.S39876 PMC3616604

[8] Newton JL Pairman J Hallsworth K Physical activity intensity but not sedentary activity is reduced in chronic fatigue syndrome and is associated with autonomic regulation. QJM 2011;104:681 7. 21382927 10.1093/qjmed/hcr029

[9] Newton J Okonkwo O Sutcliffe K Symptoms of autonomic dysfunction in chronic fatigue syndrome. QJM 2007;100:519 26. 17617647 10.1093/qjmed/hcm057

[10] Nijs J Ickmans K Postural orthostatic tachycardia syndrome as a clinically important subgroup of chronic fatigue syndrome: Further evidence for central nervous system dysfunctioning. J Intern Med 2013;273:498 500. 23331489 10.1111/joim.12034

[11] Meeus M Goubert D De Backer F Heart rate variability in patients with fibromyalgia and patients with chronic fatigue syndrome: a systematic review. Paper presented at: Seminars in Arthritis and Rheumatism; 2013. 10.1016/j.semarthrit.2013.03.004 23838093

[12] Frith J Zalewski P Klawe JJ Impaired blood pressure variability in chronic fatigue syndrome--a potential biomarker. QJM 2012;105:831 8. 22670061 10.1093/qjmed/hcs085

[13] Van Cauwenbergh D Nijs J Kos D Malfunctioning of the autonomic nervous system in patients with chronic fatigue syndrome: A systematic literature review. Eur J Clin Invest 2014;44:516 26. 24601948 10.1111/eci.12256

[14] Buchheit M Simpson M Al Haddad H Monitoring changes in physical performance with heart rate measures in young soccer players. Eur J Appl Physiol 2012;112:711 23. 21656232 10.1007/s00421-011-2014-0

[15] Lamberts RP Swart J Capostagno B Heart rate recovery as a guide to monitor fatigue and predict changes in performance parameters. Scand J Med Sci Sport 2010;20:449 57. 10.1111/j.1600-0838.2009.00977.x 19558377

[16] Nelson MJ Thomson RL Rogers DK Maximal rate of increase in heart rate during the rest-exercise transition tracks reductions in exercise performance when training load is increased. J Sci Med Sport 2014;17:129 33. 23562749 10.1016/j.jsams.2013.02.016

[17] Bellenger CR Thomson RL Howe PR Monitoring athletic training status using the maximal rate of heart rate increase. J Sci Med Sport 2015;19:590 5. 26209427 10.1016/j.jsams.2015.07.006

[18] Bellenger CR Fuller JT Thomson RL Monitoring athletic training status through autonomic heart rate regulation: a systematic review and meta-analysis. Sport Med 2016;46:1461 86. 10.1007/s40279-016-0484-2 26888648

[19] Moher D Liberati A Tetzlaff J Preferred reporting items for systematic reviews and meta-analyses: the PRISMA statement. Ann Intern Med 2009;151:264 9. 19622511 10.7326/0003-4819-151-4-200908180-00135

[20] Schardt C Adams MB Owens T Utilization of the PICO framework to improve searching PubMed for clinical questions. BMC Med Inform Decis Mak 2007;7:16. 17573961 10.1186/1472-6947-7-16 PMC1904193

[21] Sharpe M Archard L Banatvala J A report--chronic fatigue syndrome: guidelines for research. J R Soc Med 1991;84:118 21. 1999813 10.1177/014107689108400224 PMC1293107

[22] The Joanna Briggs Institute Critical Appraisal tools for use in JBI Systematic Reviews: Checklist for Case Control Studies. The Joanna Briggs Institute; 2016.

[23] Jones DEJ Hollingsworth KG Taylor R Abnormalities in pH handling by peripheral muscle and potential regulation by the autonomic nervous system in chronic fatigue syndrome. J Intern Med 2010;267:394 401. 20433583 10.1111/j.1365-2796.2009.02160.x

[24] Gibson H Carroll N Clague JE Exercise performance and fatiguability in patients with chronic fatigue syndrome, Journal of Neurology. Neurosurg Psychiatry 1993;56:993 8. 10.1136/jnnp.56.9.993 PMC489735 8410041

[25] Miwa K Fujita M Renin-aldosterone paradox in patients with myalgic encephalomyelitis and orthostatic intolerance. Int J Cardiol 2014;172:514 5. 24485613 10.1016/j.ijcard.2014.01.043

[26] Naschitz JE Rosner I Rozenbaum M The capnography head-up tilt test for evaluation of chronic fatigue syndrome. Semin Arthritis Rheum 2000;30:79 86. 11071579 10.1053/sarh.2000.9201

[27] Riley MS O’Brien CJ McCluskey DR Aerobic work capacity in patients with chronic fatigue syndrome. BMJ 1990;301:953 6. 2249024 10.1136/bmj.301.6758.953 PMC1664147

[28] Streeten DHP Streeten BW Role of impaired lower-limb venous innervation in the pathogenesis of the chronic fatigue syndrome. Am J Med Sci 2001;321:163 7. 11269790 10.1097/00000441-200103000-00001

[29] Streeten DHP Thomas D Bell DS The roles of orthostatic hypotension, orthostatic tachycardia, and subnormal erythrocyte volume in the pathogenesis of the chronic fatigue syndrome. Am J Med Sci 2000;320:1 8. 10910366 10.1097/00000441-200007000-00001

[30] Barnden LR Crouch B Kwiatek R A brain MRI study of chronic fatigue syndrome: Evidence of brainstem dysfunction and altered homeostasis. NMR Biomed 2011;24:1302 12. 21560176 10.1002/nbm.1692 PMC4369126

[31] Oosterwijck JV Marusic U De Wandele I The role of autonomic function in exercise-induced endogenous analgesia: a case-control study in myalgic encephalomyelitis/chronic fatigue syndrome and healthy people. Pain Physician 2017;20:E389 99. 28339438

[32] Cook DB Light AR Light KC Neural consequences of post-exertion malaise in Myalgic Encephalomyelitis/Chronic Fatigue Syndrome. Brain Behav Immun 2017;62:87 99. 28216087 10.1016/j.bbi.2017.02.009

[33] Hodges LD Nielsen T Baken D Physiological measures in participants with chronic fatigue syndrome, multiple sclerosis and healthy controls following repeated exercise: a pilot study. Clin Physiol Funct Imaging 2017;07:07. 10.1111/cpf.12460 28782878

[34] Vanness JM Snell CR Strayer DR Subclassifying chronic fatigue syndrome through exercise testing. Med Sci Sports Exerc 2003;35:908 13. 12783037 10.1249/01.MSS.0000069510.58763.E8

[35] Beaumont A Burton AR Lemon J Reduced cardiac vagal modulation impacts on cognitive performance in chronic fatigue syndrome. PLoS One 2012;7:e49518. 23166694 10.1371/journal.pone.0049518 PMC3498107

[36] Blackwood SK MacHale SM Power MJ Effects of exercise on cognitive and motor function in chronic fatigue syndrome and depression, Journal of Neurology. Neurosurg Psychiatry 1998;65:541 6. 10.1136/jnnp.65.4.541 PMC2170292 9771781

[37] Cook DB Nagelkirk PR Peckerman A Exercise and cognitive performance in chronic fatigue syndrome. Med Sci Sports Exerc 2005;37:1460 7. 16177595 10.1249/01.mss.0000179921.48404.ef

[38] Cook DB Nagelkirk PR Poluri A The influence of aerobic fitness and fibromyalgia on cardiorespiratory and perceptual responses to exercise in patients with chronic fatigue syndrome. Arthritis Rheum 2006;54:3351 62. 17009309 10.1002/art.22124

[39] Cordero DL Sisto SA Tapp WN Decreased vagal power during treadmill walking in patients with chronic fatigue syndrome. Clin Auton Res 1996;6:329 33. 8985621 10.1007/BF02556303

[40] De Becker P Dendale P De Meirleir K Autonomic testing in patients with chronic fatigue syndrome. Am J Med 1998;105 (3A):22S 6S. 10.1016/s0002-9343(98)00168-5 9790478

[41] De Becker P Roeykens J Reynders M Exercise capacity in chronic fatigue syndrome. Arch Intern Med 2000;160:3270 7. 11088089 10.1001/archinte.160.21.3270

[42] Farquhar WB Hunt BE Taylor JA Blood volume and its relation to peak O(2) consumption and physical activity in patients with chronic fatigue. Am J Physiol Heart Circ Physiol V 282 2002;H66 71. 10.1152/ajpheart.2002.282.1.H66 11748048

[43] Freeman R Komaroff AL Does the chronic fatigue syndrome involve the autonomic nervous system? Am J Med 1997;102:357 64. 9217617 10.1016/s0002-9343(97)00087-9

[44] Guillam̀o E Blázquez A Comella A Respiratory response to low-intensity physical exercise in women with chronic fatigue syndrome. Apunts Med l’Esport 2010;45:169 73.

[45] Hansen AL Kvale G Stubhaug B Heart rate variability and fatigue in patients with Chronic Fatigue Syndrome after a Comprehensive Cognitive Behavior group Therapy program. J Psychophysiol 2013;27:67 75.

[46] Hoad A Spickett G Elliott J Postural orthostatic tachycardia syndrome is an under-recognized condition in chronic fatigue syndrome. QJM 2008;101:961 5. 18805903 10.1093/qjmed/hcn123

[47] Hollingsworth KG Hodgson T Macgowan GA Impaired cardiac function in chronic fatigue syndrome measured using magnetic resonance cardiac tagging. J Intern Med 2012;271:264 70. 21793948 10.1111/j.1365-2796.2011.02429.x PMC3627316

[48] Hurwitz BE Coryell VT Parker M Chronic fatigue syndrome: Illness severity, sedentary lifestyle, blood volume and evidence of diminished cardiac function. Clin Sci 2010;118:125 35. 10.1042/CS20090055 19469714

[49] Ickmans K Clarys P Zinzen E Association between cognitive performance, physical fitness, and physical activity level in women with chronic fatigue syndrome. J Rehabilit Res Develop 2013;50:795 810. 10.1682/JRRD.2012.08.0156 24203542

[50] LaManca JJ Peckerman A Walker J Cardiovascular response during head-up tilt in chronic fatigue syndrome. Clin Physiol 1999;19:111 20. 10200892 10.1046/j.1365-2281.1999.00154.x

[51] LaManca JJ Peckerman A Sisto SA Cardiovascular responses of women with chronic fatigue syndrome to stressful cognitive testing before and after strenuous exercise. Psychosom Med 2001;63:756 64. 11573024 10.1097/00006842-200109000-00009

[52] Lavietes MH Bergen MT Natelson BH Measurement of CO2 in chronic fatigue syndrome patients. J Chronic Fatigue Syndr 1998;4:3 11.

[53] Miwa K Fujita M Small heart with low cardiac output for orthostatic intolerance in patients with chronic fatigue syndrome. Clin Cardiol 2011;34:782 6. 22120591 10.1002/clc.20962 PMC6652296

[54] Miwa K Down-regulation of renin-aldosterone and antidiuretic hormone systems in patients with myalgic encephalomyelitis/chronic fatigue syndrome. J Cardiol 2017;69:684 8. 27401397 10.1016/j.jjcc.2016.06.003

[55] Naschitz JE Rozenbaum M Rosner I Cardiovascular response to upright tilt in fibromyalgia differs from that in chronic fatigue syndrome. J Rheumatol 2001;28:1356 60. 11409131

[56] Naschitz JE Sabo E Naschitz S Hemodynamics Instability Score in chronic fatigue syndrome and in non-chronic fatigue syndrome. Semin Arthritis Rheum 2002;32:141 8. 12528078 10.1053/sarh.2002.34608

[57] Neary JP Roberts ADW Leavins N Prefrontal cortex oxygenation during incremental exercise in chronic fatigue syndrome. Clin Physiol Funct Imaging 2008;28:364 72. 18671793 10.1111/j.1475-097X.2008.00822.x

[58] Newton JL Sheth A Shin J Lower ambulatory blood pressure in chronic fatigue syndrome. Psychosom Med 2009;71:361 5. 19297309 10.1097/PSY.0b013e31819ccd2a

[59] Peckerman A LaManca JJ Qureishi B Baroreceptor reflex and integrative stress responses in chronic fatigue syndrome. Psychosom Med 2003;65:889 95. 14508037 10.1097/01.psy.0000079408.62277.3d

[60] Rahman K Burton A Galbraith S Sleep-wake behavior in chronic fatigue syndrome. Sleep 2011;34:671 8. 21532961 10.1093/sleep/34.5.671 PMC3079947

[61] Razumovsky AY DeBusk K Calkins H Cerebral and systemic hemodynamics changes during upright tilt in chronic fatigue syndrome. J Neuroimaging 2003;13:57 67. 12593133

[62] Robinson LJ Durham J MacLachlan LL Autonomic function in chronic fatigue syndrome with and without painful temporomandibular disorder. Fatigue Biomed Health Behav 2015;3:205 19.

[63] Schondorf R Benoit J Wein T Orthostatic intolerance in the chronic fatigue syndrome. J Auton Nerv Syst 1999;75:192 201. 10189122 10.1016/s0165-1838(98)00177-5

[64] Soetekouw PM Lenders JW Bleijenberg G Autonomic function in patients with chronic fatigue syndrome. Clin Auton Res 1999;9:334 40. 10638807 10.1007/BF02318380

[65] Suárez A Guillamó E Roig T Nitric oxide metabolite production during exercise in chronic fatigue syndrome: a case-control study. J Women's Health 2010;19:1073 7. 10.1089/jwh.2008.1255 20469961

[66] Timmers HJLM Wieling W Soetekouw PMMB Hemodynamic and neurohumoral responses to head-up tilt in patients with chronic fatigue syndrome. Clin Auton Res 2002;12:273 80. 12357281 10.1007/s10286-002-0014-1

[67] Wallman K Goodman C Morton A Test-retest reliability of the aerobic power index test in patients with chronic fatigue syndrome. J Chronic Fatigue Syndr 2003;11:19 32.

[68] Winkler AS Blair D Marsden JT Autonomic function and serum erythropoietin levels in chronic fatigue syndrome. J Psychosom Res 2004;56:179 83. 15016575 10.1016/S0022-3999(03)00543-9

[69] Yataco A Talo H Rowe P Comparison of heart rate variability in patients with chronic fatigue syndrome and controls. Clin Auton Res 1997;7:293 7. 9430800 10.1007/BF02267720

[70] Bazelmans E Bleijenberg G Van DMJ Is physical deconditioning a perpetuating factor in chronic fatigue syndrome? A controlled study on maximal exercise performance and relations with fatigue, impairment and physical activity. Psychol Med 2001;31:107 14. 11200949 10.1017/s0033291799003189

[71] Cook DB Nagelkirk PR Peckerman A Perceived exertion in fatiguing illness: Gulf War veterans with chronic fatigue syndrome. Med Sci Sport Exer 2003;35:569 74. 10.1249/01.MSS.0000058438.25278.33 12673138

[72] Cook DB Nagelkirk PR Peckerman A Perceived exertion in fatiguing illness: civilians with chronic fatigue syndrome. Med Sci Sports Exerc 2003;35:563 8. 12673137 10.1249/01.MSS.0000058360.61448.6C

[73] Fulcher KY White PD Strength and physiological response to exercise in patients with chronic fatigue syndrome, Journal of Neurology. Neurosurg Psychiatry 2000;69:302 7. 10.1136/jnnp.69.3.302 PMC1737090 10945803

[74] Inbar O Dlin R Rotstein A Physiological responses to incremental exercise in patients with chronic fatigue syndrome. Med Sci Sports Exerc 2001;33:1463 70. 11528333 10.1097/00005768-200109000-00007

[75] Nagelkirk PR Cook DB Peckerman A Aerobic capacity of Gulf War veterans with chronic fatigue syndrome. Mil Med 2003;168:750 5. 14529252

[76] Rowbottom D Keast D Pervan Z The physiological response to exercise in chronic fatigue syndrome. J Chronic Fatigue Syndr 1998;4:33 49.

[77] Sargent C Scroop GC Nemeth PM Maximal oxygen uptake and lactate metabolism are normal in chronic fatigue syndrome. Med Sci Sports Exerc 2002;34:51 6. 11782647 10.1097/00005768-200201000-00009

[78] Sisto SA LaManca J Cordero DL Metabolic and cardiovascular effects of a progressive exercise test in patients with chronic fatigue syndrome. Am J Med 1996;100:634 40. 8678084 10.1016/s0002-9343(96)00041-1

[79] Strahler J Fischer S Nater UM Norepinephrine and epinephrine responses to physiological and pharmacological stimulation in chronic fatigue syndrome. Biol Psychol 2013;94:160 6. 23770415 10.1016/j.biopsycho.2013.06.002

[80] Jones DEJ Hollingsworth KG Jakovljevic DG Loss of capacity to recover from acidosis on repeat exercise in chronic fatigue syndrome: A case-control study. Eur J Clin Invest 2012;42:186 94. 21749371 10.1111/j.1365-2362.2011.02567.x

[81] Paul LM Wood L Maclaren W The effect of exercise on gait and balance in patients with chronic fatigue syndrome. Gait Posture 2001;14:19 27. 11378421 10.1016/s0966-6362(00)00105-3

[82] Beaver WL Wasserman K Whipp BJ Improved detection of lactate threshold during exercise using a log-log transformation. J Appl Physiol 1985;59:1936 40. 4077801 10.1152/jappl.1985.59.6.1936

[83] Shu Q Wang H Litscher D Acupuncture and Moxibustion have Different Effects on Fatigue by Regulating the Autonomic Nervous System: A Pilot Controlled Clinical Trial. Sci Rep 2016;6:37846. 27886247 10.1038/srep37846 PMC5122953

[84] Sisto SA Tapp W Drastal S Vagal tone is reduced during paced breathing in patients with the chronic fatigue syndrome. Clin Auton Res 1995;5:139 43. 7549414 10.1007/BF01826195

[85] Togo F Natelson BH Heart rate variability during sleep and subsequent sleepiness in patients with chronic fatigue syndrome. Auton Neurosci 2013;176:85 90. 23499514 10.1016/j.autneu.2013.02.015 PMC4100066

[86] Lavietes MH Natelson BH Cordero DL Does the stressed patient with chronic fatigue syndrome hyperventilate? Auton Neurosci 1996;3:70 83. 10.1207/s15327558ijbm0301_6 16250768

[87] Gallagher AM Coldrick AR Hedge B Is the chronic fatigue syndrome an exercise phobia? A case control study. J Psychosom Res 2005;58:367 73. 15992572 10.1016/j.jpsychores.2005.02.002

[88] Robinson B Epstein S Beiser GD Control of heart rate by the autonomic nervous system: studies in man on the interrelation between baroreceptor mechanisms and exercise. Circ Res 1966;19:400 11. 5914852 10.1161/01.res.19.2.400

[89] Jose AD Taylor RR Autonomic blockade by propranolol and atropine to study intrinsic myocardial function in man. J Clin Invest 1969;48:2019 31. 5398888 10.1172/JCI106167 PMC297454

[90] Hirsch JA Bishop B Respiratory sinus arrhythmia in humans: how breathing pattern modulates heart rate. Am J Physiol V 241 1981;H620 9. 10.1152/ajpheart.1981.241.4.H620 7315987

[91] Sime WE Whipple IT Berkson DM Reproducibility of heart rate at rest and in response to submaximal treadmill and bicycle ergometer test in middle-aged men. Med Sci Sports Exerc 1972;4:14 7. 5020477

[92] Akselrod S Gordon D Ubel FA Power spectrum analysis of heart rate fluctuation: a quantitative probe of beat-to-beat cardiovascular control. Science 1981;213:220 2. 6166045 10.1126/science.6166045

[93] Otzenberger H Gronfier C Simon C Dynamic heart rate variability: a tool for exploring sympathovagal balance continuously during sleep in men. Am J Physiol 1998;275:H946 50. 9724299 10.1152/ajpheart.1998.275.3.H946

[94] Thayer JF Yamamoto SS Brosschot JF The relationship of autonomic imbalance, heart rate variability and cardiovascular disease risk factors. Int J Cardiol 2010;141:122 31. 19910061 10.1016/j.ijcard.2009.09.543

[95] Stewart J Weldon A Arlievsky N Neurally mediated hypotension and autonomic dysfunction measured by heart rate variability during head-up tilt testing in children with chronic fatigue syndrome. Clin Auton Res 1998;8:221 30. 9791743 10.1007/BF02267785

[96] Duprez DA De Buyzere ML Drieghe B Long-and short-term blood pressure and RR-interval variability and psychosomatic distress in chronic fatigue syndrome. Clin Sci 1998;94:57 63. 9505867 10.1042/cs0940057

[97] Chidsey CA Braunwald E Sympathetic activity and neurotransmitter depletion in congestive heart failure. Pharmacol Rev 1966;18:685 700. 5904181

[98] Howley ET Bassett DR Welch HG Criteria for maximal oxygen uptake: review and commentary. Med Sci Sports Exerc 1995;27:1292 301. 8531628

[99] Van Oosterwijck J Nijs J Meeus M Pain inhibition and postexertional malaise in myalgic encephalomyelitis/chronic fatigue syndrome: an experimental study. J Intern Med 2010;268:265 78. 20412374 10.1111/j.1365-2796.2010.02228.x

[100] Tanaka H Monahan KD Seals DR Age-predicted maximal heart rate revisited. J Am Coll Cardiol 2001;37:153 6. 11153730 10.1016/s0735-1097(00)01054-8

[101] Andreacci JL Lemura LM Cohen SL The effects of frequency of encouragement on performance during maximal exercise testing. J Sport Sci 2002;20:345 52. 10.1080/026404102753576125 12003280

[102] Nijs J Meeus M Heins M Kinesiophobia, catastrophizing and anticipated symptoms before stair climbing in chronic fatigue syndrome: An experimental study. Disabil Rehabil 2012;34:1299 305. 22324510 10.3109/09638288.2011.641661

[103] Bou-Holaigah I Rowe PC Kan J The relationship between neurally mediated hypotension and the chronic fatigue syndrome. J Am Med Assoc 1995;274:961 7. 7674527

[104] Stewart JM Autonomic nervous system dysfunction in adolescents with postural orthostatic tachycardia syndrome and chronic fatigue syndrome is characterized by attenuated vagal baroreflex and potentiated sympathetic vasomotion. Pediatr Res 2000;48:218 26. 10926298 10.1203/00006450-200008000-00016

[105] Weise F Heydenreich F Runge U Contributions of sympathetic and vagal mechanisms to the genesis of heart rate fluctuations during orthostatic load: a spectral analysis. J Autonom Nerv Sys 1987;21:127 34. 10.1016/0165-1838(87)90015-4 3450691

[106] De Lorenzo F Xiao H Mukherjee M Chronic fatigue syndrome: physical and cardiovascular deconditioning. QJM 1998;91:475 81. 9797930 10.1093/qjmed/91.7.475

[107] Nijs J De Meirleir K Duquet W Kinesiophobia in chronic fatigue syndrome: assessment and associations with disability. Arch Phys Med Rehabil 2004;85:1586 92. 15468015 10.1016/j.apmr.2003.12.033

[108] Miwa K Fujita M Small heart syndrome in patients with chronic fatigue syndrome. Clin Cardiol 2008;31:328 33. 18636530 10.1002/clc.20227 PMC6653127

[109] Maron BJ Pelliccia A Spataro A Reduction in left ventricular wall thickness after deconditioning in highly trained Olympic athletes. Br Heart J 1993;69:125 8. 8435237 10.1136/hrt.69.2.125 PMC1024938

[110] Rossy LA Thayer JF Fitness and gender-related differences in heart period variability. Psychosom Med 1998;60:773 81. 9847039 10.1097/00006842-199811000-00022

[111] Carter JB Banister EW Blaber AP Effect of endurance exercise on autonomic control of heart rate. Sports Med 2003;33:33 46. 12477376 10.2165/00007256-200333010-00003

[112] Lamb L Johnson R Stevens P Cardiovascular deconditioning during chair rest. Aerosp Med 1964;35:646 9. 14177466

[113] Londeree B Effect of training on lactate/ventilatory thresholds: a meta-analysis. Med Sci Sports Exerc 1997;29:837 43. 9219214 10.1097/00005768-199706000-00016

[114] Burke J Thayer R Belcamino M Comparison of effects of two interval-training programmes on lactate and ventilatory thresholds. Br J Sports Med 1994;28:18 21. 8044486 10.1136/bjsm.28.1.18 PMC1332151

[115] Jones AM Carter H The effect of endurance training on parameters of aerobic fitness. Sports Med 2000;29:373 86. 10870864 10.2165/00007256-200029060-00001

[116] Gladden LB Yates J Stremel RW Gas exchange and lactate anaerobic thresholds: inter-and intraevaluator agreement. J Appl Physiol 1985;58:2082 9. 4008424 10.1152/jappl.1985.58.6.2082

[117] Åstrand P-O Ryhming I A nomogram for calculation of aerobic capacity (physical fitness) from pulse rate during submaximal work. J Appl Physiol 1954;7:218 21. 13211501 10.1152/jappl.1954.7.2.218

[118] Karvonen J Vuorimaa T Heart rate and exercise intensity during sports activities. Sports Med 1988;5:303 11. 3387734 10.2165/00007256-198805050-00002

[119] Meeus M Nijs J Central sensitization: a biopsychosocial explanation for chronic widespread pain in patients with fibromyalgia and chronic fatigue syndrome. Clin Rheumatol 2007;26:465 73. 17115100 10.1007/s10067-006-0433-9 PMC1820749

[120] van der Werf SP Prins JB Vercoulen JH Identifying physical activity patterns in chronic fatigue syndrome using actigraphic assessment. J Psychosom Res 2000;49:373 9. 11164063 10.1016/s0022-3999(00)00197-5

[121] Lamberts R Lambert M Day-to-day variation in heart rate at different levels of submaximal exertion: Implications for monitoring training. J Strength Cond Res 2009;23:1005 10. 19387374 10.1519/JSC.0b013e3181a2dcdc

[122] VanNess JM Stevens SR Bateman L Postexertional malaise in women with chronic fatigue syndrome. J Women Health 2010;19:239 44. 10.1089/jwh.2009.1507 20095909

